# Molecular Biology in the Improvement of Biological Nitrogen Fixation by Rhizobia and Extending the Scope to Cereals

**DOI:** 10.3390/microorganisms9010125

**Published:** 2021-01-07

**Authors:** Ravinder K. Goyal, Maria Augusta Schmidt, Michael F. Hynes

**Affiliations:** 1 Lacombe Research and Development Centre, Agriculture and Agri-Food Canada, Lacombe, AB T4L 1W1, Canada; augusta.schmidt@canada.ca; 2 Department of Biological Sciences, University of Calgary, 2500 University Dr NW, Calgary, AB T2N 1N4, Canada; hynes@ucalgary.ca

**Keywords:** biological nitrogen fixation, efficiency, molecular biology, rhizobia, stress, ethylene, nodulation, improvement, cereals

## Abstract

The contribution of biological nitrogen fixation to the total N requirement of food and feed crops diminished in importance with the advent of synthetic N fertilizers, which fueled the “green revolution”. Despite being environmentally unfriendly, the synthetic versions gained prominence primarily due to their low cost, and the fact that most important staple crops never evolved symbiotic associations with bacteria. In the recent past, advances in our knowledge of symbiosis and nitrogen fixation and the development and application of recombinant DNA technology have created opportunities that could help increase the share of symbiotically-driven nitrogen in global consumption. With the availability of molecular biology tools, rapid improvements in symbiotic characteristics of rhizobial strains became possible. Further, the technology allowed probing the possibility of establishing a symbiotic dialogue between rhizobia and cereals. Because the evolutionary process did not forge a symbiotic relationship with the latter, the potential of molecular manipulations has been tested to incorporate a functional mechanism of nitrogen reduction independent of microbes. In this review, we discuss various strategies applied to improve rhizobial strains for higher nitrogen fixation efficiency, more competitiveness and enhanced fitness under unfavorable environments. The challenges and progress made towards nitrogen self-sufficiency of cereals are also reviewed. An approach to integrate the genetically modified elite rhizobia strains in crop production systems is highlighted.

## 1. Introduction

Biological nitrogen fixation (BNF) was the primary source of converting atmospheric nitrogen into a usable organic form until the Haber–Bosch process was discovered in the early 20th century and commercialized since then. Fueled by a seemingly unlimited supply of hydrocarbons, this chemical process provided an easily accessible source of N fertilizers, which played a central role in the crop productivity revolution. During the past century, chemical fertilizers witnessed an exponential growth of synthesis and consumption to the point that the contribution of BNF in crop productivity became obscure. N fertilizer use in cereal and other crops became a routine part of the agricultural practices and farmers were even tempted to supplement the nitrogen in legume crops. Undoubtedly, N fertilizers were paramount in providing a food security cover which otherwise would have been an uphill task at the existing productivity level [1]. In the past, intensive use of the factory-produced fertilizers was less of an environmental concern. A high carbon footprint and nitrogen pollution of water bodies such as eutrophication [2] have provided enough reason to pause and question the sustainability of the steep upward curve of N fertilizer usage. Due to environmental concerns regarding the synthetic option and the additional cost of cultivation, the significance of BNF has re-gained focus in agriculture production systems.

The contribution of BNF in agricultural systems ranges from 40 to 70 Tg N y^−1^ [3,4], which is approximately at 50% of global production of N fertilizers. More nitrogen will be needed in pursuit of achieving higher agricultural productivity goals to feed a growing population. Improvements in BNF can play an important role in bridging the supply/demand gap and reducing dependence on the chemical route. There are many areas where the improvements can be made to increase output of fixed nitrogen. The efficiency and efficacy of the rhizobia could play a key role in determining the output. The former relates to the catalytic efficiency of nitrogenase using host nutrients, whereas efficacy indicates how well rhizobia can perform under the given conditions [5]. Several factors adversely affect the survival and performance of rhizobia, with negative consequences on nitrogen fixation. Many of these factors are extremes of environments and unfavorable soil conditions. Extensive efforts have been made to search for microorganisms that have improved efficiency of BNF, are adapted to the extremes of environments, and are more competitive in a microbiome sphere. The identification of such strains, which are specific to the conditions, presents opportunities to study the adaptive mechanisms. Recombinant DNA technology has provided powerful tools to manipulate the mechanisms and transfer desirable traits to the strains that are better in other symbiotic characteristics.

Cereals such as wheat, rice and maize are consumed as staple foods, providing 75% of the world’s calorie uptake [6]. They dominate in the acreage of total arable land compared to leguminous crops. There was only 3.3%, 12.5% and 32% of grain legume crops in the total area of arable crops in EU, Canada and USA, respectively, in 2006 [7]. Rhizobia do not forge a symbiotic relationship with cereals. Except some contribution from associative symbiosis and free-living nitrogen-fixing bacteria, most of the nitrogen demand is met through synthetic N fertilizer to maintain the optimum productivity level. Cereal crops, therefore, are the principal contributing factor to the overall consumption of synthetic versions of nitrogen. Enabling cereals to fix their own nitrogen could be a leap forward in sustainable agriculture. Deploying various strategies, attempts have been made in recent decades to explore the possibility of nitrogen self-sufficiency in cereals.

The process of nitrogen fixation through symbiosis is very complex, involving multiple events and their regulation in both the host and the rhizobia. This makes the task of developing symbiosis in cereals very difficult, especially considering the fact that the process did not evolve naturally over a long period of time. Nevertheless, a series of steps in symbiosis provide numerous opportunities to optimize them for increasing the productivity of BNF. Molecular biology has played a significant role in decoding the symbiotic mechanisms and applying the knowledge both in strain improvement and creating new possibilities. The progress achieved in this area of BNF has been reviewed here.

## 2. Improvement of Rhizobial Strains

To maximize the amount of nitrogen conversion by the rhizobia, different stages of the plant–bacteria interaction could be optimized. The chemical communication between the bacteria and its host plant can be improved to enhance the number of nodules formed and favor occupation by desirable strains. Flavonoids produced by the plant are the earliest signals that promote the symbiotic interaction, and induce the genes (*nod* genes) that are responsible for synthesis of Nod factors. Chemically, these are Lipochitooligosaccharides that signal the plant to initiate nodule formation [8]. Manipulation of *nod* genes, especially for their expression under suboptimal environmental conditions can significantly enhance symbiotic efficiency [9]. For example, increasing Nod factor availability at low soil temperatures can enhance nodulation [10,11], though in some specific cases an excess of Nod factor can block nodulation of primitive cultivars [12]. Nitrogen reduction could be made more efficient by facilitating the transport of electrons in a reaction catalyzed by the nitrogenase. Overexpression of genes from *nif* and *fix* groups is being used to develop such technologies [13,14,15,16,17,18]. Modulation of stress response genes, phytohormone biosynthesis, phosphate solubilization, and antibiotic production are among the other areas of symbiotic interaction that are being explored to enhance rhizobia effectiveness [19]. Table 1 summarizes the molecular modifications in rhizobial strains and its effect on host–bacteria symbiotic processes. Prominent areas with significant developments are discussed.

### 2.1. The Enhanced Efficiency of Nitrogen Fixation

The rhizobial strains could be improved at any stage beginning with their perception of flavonoids to initiate the nodulation process, through all the steps up to delivery of fixed nitrogen to the host plant. Symbiotic nitrogen fixation in rhizobia is mostly controlled by *nod*, *nif* and *fix* genes [5]. Changing the *nodD* gene expression from an induced to a constitutive state to broaden the host range of symbiosis in tropical legumes was among the earliest approaches to exploit the potential of DNA recombinant technology [41]. The DNA fragment harboring the *nodD1* gene, the common nodulation genes (*nodABC*), and an operon essential for nitrogen fixation (*nifN*) from the *nod* regulon region of *S. meliloti* was integrated to generate average 2.5 copies of the region [40]. The derivative strain significantly improved symbiotic properties, with increased nodulation, nitrogenase activity and overall growth in alfalfa. The modifications in *nif* genes and the potential benefit of improved strains were tested under field conditions. In a study, *S. meliloti* was engineered for extra copies of *nifA* and *dctABD* genes and the modified strain resulted in an approximately 13% increase in alfalfa biomass yield [16]. The *dctABD* genes encode a dicarboxylate transport system (and its regulatory genes) responsible for the import of the carbon compounds as a source of energy which is crucial to support the function of nitrogenase [81]. Engineering nitrogen reductase (*nifH*) of *R. etli* as a *nifHDK* operon under the *nifHc* promoter that had only a truncated version of the operon resulted in enhanced nitrogenase activity (58% on average), increased plant weight (32% on average), increased nitrogen content and seed yield (36%) in common bean plants [17]. In addition, a genetic modification in poly-beta-hydroxybutyrate-negative background further enhanced the symbiotic efficiency of the recombinant strain. The studies on adding extra copies or restoring the full functionality of existing copies of nitrogenase components leading to a positive impact on the nitrogenase function suggest the latter to be a limiting factor in nitrogen fixation. The beneficial effect of protein engineering to increase the catalytic function of nitrogenase was demonstrated successfully a few decades ago [82]. With better understanding of nitrogenase structure and function, progress has been made in improving the enzyme’s catalytic function [83,84]. In nitrogen fixation reactions, H_2_ is produced as a by-product, which, in most of rhizobial strains, diffuses into the environment. It is not clearly known why approximately 25% of the total energy consumption is wasted in an otherwise energy expensive reaction [85]. There exist some strains that have the ability to metabolize H_2_ through uptake hydrogenases (Hup). These strains are referred to as Hup^+^ and those that do not have this activity are termed as Hup^-^ strains. Could the transfer of the hydrogenase gene to Hup^-^ strains improve symbiotic efficiency? The gain in symbiotic efficiency has been demonstrated by Torres and coworkers who transferred an 18 kb *hup* gene cluster from *R. leguminosarum* bv. *viciae* encoding a NiFe hydrogenase to a common bean rhizobial strain lacking hydrogenase [27,86]. However, other studies [87] have found no increase in nitrogen fixation in pea after adding *hup* genes. Regulation of *hup* genes is complex and heavily influenced by host plant background and nickel availability, so these conflicting results are not surprising [88]; there is a strong consensus that in soybean symbionts, uptake hydrogenase systems do enhance nitrogen fixation and crop yield [89]. Due to the sensitivity of nitrogenase to oxygen, a low concentration environment is maintained in bacteroids. However, oxygen is required in the electron transport chain to generate energy to support nitrogen reduction. To overcome this challenge, bacteria synthesize a high-affinity cytochrome cbb3 oxidase [90]. Overexpression of this oxidase in *R. etli* was associated with higher symbiotic performance [50]. Other strategies that are not directly targeted at nitrogenase have also been employed to improve the symbiotic efficiency. In one study, additional copies of the *clpB* gene that encodes a chaperone protein were transferred to *M. mediterraneum* and a symbiotic analysis was performed with chickpea [25]. In addition to conferring tolerance to stress, the transformed strain induced a greater number of nodules and increased symbiotic effectiveness by 60–80% depending on the pH conditions. The higher nodulation was correlated with enhanced expression of nodulation genes *nodA* and *nodC*. Chaperone proteins and other stress-responsive genes that play an important role in symbiosis have been comprehensively reviewed by da-Silva et al. [26]. Reduction in oxidative stress through enhanced expression of catalase, which effectively lowered the H_2_O_2_ content, led to an approximately 2-fold increase in nitrogen fixation [52]. A similar increase in symbiont performance was demonstrated with extra copies of the *ahpC* gene that lowered the peroxide, superoxide and malondialdehyde contents in *Anabaena* strains [53]. For more details on the role of redox status in regulation of plant and bacterial differentiation and the symbiotic process, the readers are referred to a review [91]. The secretion of exopolysaccharide can also improve symbiotic efficiency, which has been demonstrated by overexpressing the *exoY* gene in a *S. meliloti* strain, enabling increased production of succinoglycan [24]. The modified strain enhanced the symbiotic productivity in *M. truncatula*. Regulation of assimilation of fixed nitrogen through distribution and allocation of nitrate in the plant parts has been explored as another strategy to improve symbiotic performance. In plants, the nitrogen flux across different organs is controlled by transporters that belong to mainly four protein families, namely chloride channels, slowly activating anion channels, nitrate/peptide transporters (NPF) and nitrate transporters [92]. By disrupting *L. japonicus* transporter LjNPF2.9, Sol et al. [93] demonstrated an increase in shoot biomass without affecting symbiotic nitrogen fixation. In summary, the strains were genetically modified targeting various genes and different processes of host–rhizobial symbiosis. The improvement resulting from these modifications suggests that a variety of factors determine the symbiotic performance and yet there might be more areas of host–rhizobial interaction that can be optimized in terms of increasing amount of N_2_ fixed.

BNF in nodules is not only controlled by the symbiont but the host plant also plays an important role. Because the latter is responsible for supply of carbon that supports nitrogenase activity, the nodule development, number and nitrogen turnover are subject to regulation by the host [94]. A higher rate of nitrogen fixation by an improved symbiont will still be dependent on the regulatory constraints of carbon and nitrogen metabolism in the host plant. There is a high demand of Pi in metabolically active nodules [95]. Increasing the nodule number to achieve more N output could have a negative impact on plant growth and development, if there is a phosphorus deficiency. Therefore, it is not necessary that positive results of every strategy will translate into measurable gains in different situations. For example, the success of hup^+^-modified strains can only be ascertained in field conditions, especially when H_2_ is known to promote plant growth-promoting rhizobacteria that allow certain plants to compete successfully early in the growing season [96].

### 2.2. Microsymbiont Competitiveness

One of the obstacles to widespread use of superior strains of rhizobia, irrespective of whether these strains are selected from natural populations or engineered to include beneficial attributes, is competition from soil microflora, including native strains of rhizobia that can form nodules on the same crop. Indigenous strains are usually found anywhere there is a history of cultivation of a given legume species, and can arise from horizontal gene transfer from inoculant strains to non-symbiotic bacteria present in soils [97]. The native strains are usually well adapted to local soil conditions but inefficient at fixing nitrogen, yet they will form the bulk of nodules on a crop because of their superior competitiveness; this has been termed “the rhizobium competition problem” [98].

Solutions to this competition problem require an in-depth understanding of the attributes of individual rhizobial strains that contribute to their competitiveness for formation of nodules, and ability to survive and proliferate in the rhizosphere. These include motility and chemotaxis [99,100,101], production of antibiotics and bacteriocins (discussed below), and ability to catabolize a variety of different carbon sources, some of which are known to be specifically secreted by plants.

The capacity to take up and metabolize many sugars, sugar alcohols, amino acids, and other compounds has been shown to affect the ability of rhizobial strains mutated in these processes to compete against isogenic wild-type strains. For example, this has been shown for rhamnose [102,103], proline [104], erythritol [105], and glycerol [106], although the ability to use these carbon and energy sources is fairly widespread in rhizobia, and probably of little value in terms of manipulating bacteria. More specific compounds such as the non-protein amino acid homoserine, which is produced in large amounts by pea seedlings [107], and rhizopines [108] offer better possibilities for engineering strains suited to specific rhizospheres. Homoserine catabolism appears to be restricted to isolates adapted to pea rhizospheres [109,110], and it has been shown that the ability to transport and catabolize homoserine is necessary for competitive nodulation [110].

The rhizopines, which are rare scyllo-inosamine derivatives synthesized by rhizobia themselves in nodules [108,111,112], and catabolized by the free-living rhizobia, offer an intriguing possibility for manipulation of the rhizosphere to give a competitive advantage only to certain selected strains. Proof of principle for this was provided by Geddes et al. [113], who engineered *M. truncatula* and barley plants to synthesize rhizopines. These plants were able to induce expression of rhizopine catabolic gene promoters in bacteria in the root region, and select for increased populations of engineered rhizopine catabolizing rhizobia. Creating a biased rhizosphere in this fashion has the potential to eliminate competition from native strains.

Antimicrobial compounds produced by rhizobia vary greatly among strains, and have been examined for their role in competition. Bacteriocins are narrow-spectrum antibiotics that are usually proteinaceous in nature [114], and several different rhizobial bacteriocins have been shown to play some role in promoting competitive nodulation [115,116]. The biggest success story in terms of antimicrobial production is trifolitoxin, a small-peptide antibiotic/bacteriocin that is made by only a few strains of *R. leguminosarum* [117,118]. Engineered strains producing trifolitoxin have been shown to have greatly enhanced competitiveness in both lab and field trials [21,119]. This ability could potentially be exploited further in design of strains of many species of rhizobia with enhanced competitiveness.

Another approach to solving the competition problem may be to select for or design bacteriophage-resistant inoculant strains with superior symbiotic attributes. These can be co-inoculated onto seeds with a cocktail of phages that target native strains and eliminate them as competitors. Several studies with phage-resistant strains have proven that this approach has validity [120,121]. Further isolation and characterization of phages that are lytic for rhizobia, and of rhizobial phage defense mechanisms will be required to exploit this approach on a wider scale.

### 2.3. Stress Tolerance

For the strains to perform efficiently under environmental stress and to maintain their competitiveness, they should have adequate tools to mitigate the stress. The geographical distribution of strains as a result of their adaptation to specific conditions suggests the presence of certain mechanisms enabling their survival and functionality. Such mechanisms provide resources and opportunities to improve strains with high symbiotic performance which otherwise are not capable of withstanding a particular stress. Genetic improvements of strains conferring tolerance against a variety of stresses have been achieved through recombinant DNA technology. It is important to keep in mind that there will be challenges in converting these strains into commercial opportunities due to competition with the native strains. The studies indicate that commercial strains do not always outperform the native interactions [122,123].

#### 2.3.1. Drought and Salinity

Though drought and salinity are different types of stresses, there is, in both cases, a reduced availability of water. Often, the perception and tolerance mechanisms of these two types of stresses display many similarities. The accumulation of osmoprotectants such as sugars, polyols, betaines, and amino acids is one of the responses under stress to maintain membrane structure and cellular function. Overexpression of trehalose-6-phosphate synthase, which catalyzes a step towards trehalose accumulation, in the symbiotic bacterium *R. etli* improved the strain performance under drought measured by nitrogenase activity, growth and biomass of *P. vulgaris* [76]. The study suggested that trehalose was not only an osmoprotectant but also played a role in a signaling mechanism for plant growth and adaptation to the stress. The importance of trehalose accumulation in stress tolerance was further documented in the chickpea—*M. ciceri* symbiosis. The overexpression of the gene *otsA* that encodes trehalose-6-phosphate synthase in the rhizobial strain enabled the host to form more nodules and higher shoot biomass than wild-type strains under salinity stress [77]. Whereas in the previous studies the enhanced de novo synthesis of an osmolyte was targeted, in another study the impact of increased uptake of such molecules was studied. BetS is a high-affinity glycine and proline betaine uptake system involved in the rapid acquisition of betaines by cells under osmotic stress. Boscari et al. [75] overexpressed this transporter, resulting in 2.3-fold higher glycine betaine transport than in the wild-type strain. In the host plant alfalfa, there was an approximately 40% increase in proline betaine accumulation under salinity stress. The nodules with the overexpressing strain maintained a superior nitrogenase function to the wild type. A higher accumulation of the osmolytes proline and pinitol was among the metabolic changes observed in alfalfa due to overproduction of IAA in the symbiotic *Rhizobium* strain [63]. The host plant showed better adaptation to drought conditions upon forming symbiosis with the IAA-overproducing strain and there was downregulation of ABA biosynthesis genes. Increase in ABA is a characteristic indicator of drought stress status in plants. Induction of stress-responsive genes upon IAA treatment was observed through transcriptomic profiling in *B. japonicum*, suggesting that IAA is a positive regulator of stress tolerance [124]. Most of the abiotic stresses are associated with changes in redox environment causing oxidative stress, possibly acting as a signal for downstream metabolic changes [125,126,127]. The prevention of oxidative stress through flavonoids was used as a strategy to mitigate salinity stress in the alfalfa—*S. meliloti* symbiosis [46]. The flavonoid-overproducing strain reduced the salt-induced structural damage and had a protective effect on the nodule structure and function. Enhanced tolerance to drought stress was achieved through enhancing the function of cbb-3 oxidase in *R. etli* that displayed higher respiratory activity under stress [51]. Cytochrome cbb3 oxidase is a member of the heme-copper oxidase superfamily that catalyzes an oxidation reaction and coupled pumping of protons.

#### 2.3.2. Heat Stress

Extreme high temperature limits the geographical distribution of strains and their ability to nodulate, depriving many crops of the benefit of symbiotic nitrogen fixation [128,129]. An improvement in heat tolerance can not only restore the nodulating ability of strains under conditions which otherwise are non-conducive to normal symbiotic interaction, but also enhance strain survival in hostile temperatures. Efforts have been made to achieve the objective through molecular manipulations. The chaperones, many of which are activated during stress, play an important role in stress tolerance or adaptation [130]. Knocking out a ClpB chaperone in a *M. ciceri* strain resulted in sensitivity to heat stress [131]. There was a delayed appearance of nodules and a greater proportion of them were ineffective. Conversely, additional copies of this chaperone gene provided more tolerance to heat stress, with overall higher symbiotic effectiveness [25]. The effectiveness of other chaperone genes in heat stress tolerance has also been observed. Additional copies of *groEL* in a *Mesorhizobium* strain allowed enhanced recovery from heat shock of 48 °C for 15 min [26]. In addition, the modified strain displayed a greater symbiotic effectiveness.

#### 2.3.3. Metal Toxicity

Industrialization greatly helped in alleviating poverty but it came at an environmental cost, especially in the case of heavy metal toxicity in areas exposed to industrial waste [132]. Soil polluted with heavy metals not only restricts plant productivity, but also poses an increased risk to human health due to bioaccumulation of toxic contaminants. In bioremediation strategies, both plants with tolerance to metal stress and microorganisms with metal scavenging or detoxifying activity could be deployed. Due to the presence of a wide range of mechanisms, microorganisms serve as a powerful tool to mitigate the metal stress [133,134]. Symbiotic nitrogen fixation is limited to a select category of microorganisms, which may not necessarily have highly adapted mechanisms for tolerating and detoxifying metals, but through genetic manipulation success has been achieved against several metals. Inoculation with *S. medicae* expressing copper resistance genes, *copAB* from *P. fluorescens* was able to phytostabilize the toxic level of Cu (copper) in *M. truncatula* [31]. Further improvement in Cu tolerance was observed when the host plant was also modified with a metallothionein gene *mt4a* and inoculated with *S. medicae* [32]. In another study, Cu tolerance by *R. etli* was enhanced upon deletion of a plasmid carrying *ropAe* gene encoding an outer membrane protein with a β-barrel channel structure that likely facilitates Cu transport [34]. Cadmium (Cd), which finds its way into the environment through mining, smelting and municipal wastes becomes a source of exposure with adverse effects on human health (https://www.atsdr.cdc.gov/csem/csem.asp?csem=6&po=4). For bioremediation against Cd, separately, two recombinant *M. huakuii* strains, each expressing a synthetic tetrameric metallothionein (MTL4) and phytochelatin synthase from *A. thaliana* (AtPCS), were constructed [28]. The inoculation of *Astragalus sinicus* plants with the modified strains led to substantial accumulation of Cd in both nodules and roots. The expression of another iron-regulated transporter from *Arabidopsis* (AtIRT1) enabled *M. huakuii* strains to accumulate increased amounts of Cu and arsenic (As) in the nodules [29]. Arsenite bioremediation was also demonstrated through its methylation by S-adenosylmethionine methyltransferase [33]. The researchers expressed the gene from *C. reinhardtii* in a *R. leguminosarum* bv. *trifolii* strain and a sizeable amount of methylation could be achieved in red clover with recombinant *Rhizobium* symbiosis. Oxidation of arsenite can be another mechanism of improved tolerance as observed in *S. meliloti* where pSinA plasmid was found to carry the genes responsible for oxidation [30]. The effectiveness of metal efflux pumps in the host plant has been shown by expressing arsenite efflux pump Acr3 from *S. medicae* in tobacco plants [135]. It was noted that targeting of Acr3 to the tonoplast was more effective than its expression on the plasma membrane. The importance of metallothionein proteins in amelioration of heavy metal toxicity is further underlined in a more recent study. Two recombinant strains of *R. leguminosarum* bv. *Viciae*, each expressing a pea metallothionein gene, *PsMT1* or *PsMT2*, led to normal development of nodules in pea under Cd stress [35]. Because the heavy metals cause oxidative stress [136], the other strategies could be based on maintaining the homeostasis of metals through different mechanisms [133]. The observed tolerance to copper in *M. lupulina* by ACC deaminase-overproducing *S. meliloti* suggests that stress signaling interference could also lead to resistance against metal toxicity [68]. It remains to be seen whether a mitigation strategy for one heavy metal will also hold true for other heavy metals.

## 3. Reduction in Ethylene Synthesis and Nodulation

**Figure 1:**
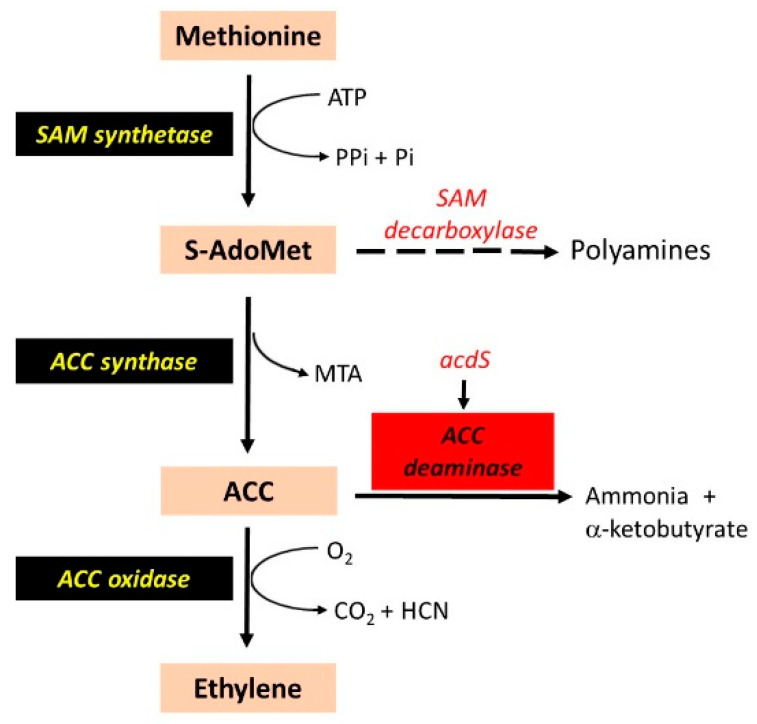
Pathway of ethylene synthesis and reduction in its level through substrate mobilization. S-AdoMet or SAM, S-adenosylmethionine; MTA, Methylthioadenosine; ACC, 1-aminocyclopropane-1-carboxylic acid.

Ethylene, initially recognized as a fruit ripening hormone, is involved in multiple aspects of plant growth and development. Ethylene interacts with other hormones to modulate disease and abiotic stress response and plays a major role in senescence. Ethylene is a negative regulator of nodulation, although its role in early symbiotic events has been recognized [137,138]. Interference in ethylene signaling through application of aminoethoxyvinylglycine stimulated nodulation in alfalfa [139]. Further, several mutants and transgenic plants altered in ethylene level and signaling showed variation in nodule size and number [137]. The control of ethylene synthesis and perception, thus, presents opportunities to enhance the nodulating ability of *Rhizobium*. In addition to nodulation, the manipulation of ethylene response can also have a positive effect on abiotic stress tolerance. Ethylene is synthesized from its immediate precursor 1-aminocyclopropane-1-carboxylic acid (ACC) through oxidation, a reaction catalyzed by ACC oxidase. Because under normal conditions this reaction is not a rate-limiting step, the level of ACC directly impacts the ethylene level [140]. ACC can be metabolized through its deamination by ACC deaminase, which is encoded by the *acdS* gene (Figure 1).

Among a collection of *B. japonicum* isolates, those expressing higher level of *acdS* had higher numbers of nodules, biomass and delayed senescence of the nodules [141]. Overexpression of *acdS* in *M. ciceri* increased nodulation in chickpea, improved the growth and plant biomass, reduced susceptibility to root-rot disease and improved tolerance to waterlogged conditions [66,67]. Similarly, the exogenous expression of *acdS* in other species of *Mesorhizobium* increased chickpea tolerance against salinity [68]. A higher plant biomass compared to a wild-type strain was obtained under stress in *M. lupulina* by inoculating with ACC deaminase-overproducing *S. meliloti* transformants [69]. Increased nodulation due to overexpression of ACC deaminase was earlier observed in alfalfa and pea [56,57]. The effect of ACC deaminase on nodulation was also exerted by the co-inoculants along with *Rhizobium*. The exogenous expression of ACC deaminase in *Serratia grimesii* BXF1 and its co-inhabitation with *R. tropici* led to an early nodulation in common bean [142]. Similarly, co-inoculation of plant growth-promoting bacteria containing ACC deaminase with *Rhizobium phaseoli* strains enhanced plant growth and induced salt tolerance in *Vigna mungo* [143].

## 4. Nitrogen Fixation in Cereals

In view of the overwhelming benefits, the idea of enabling cereal crops to fix their own nitrogen with little or no reliance on external input has fascinated the scientific community in recent decades. A tremendous advancement in the field notwithstanding [144,145,146], the task has been extremely challenging. The recent developments in the field have been discussed here.

It is important to assess the magnitude of the challenge in making the cereals nitrogen self-sufficient before different strategies and progress in that pursuit are discussed. Atmospheric nitrogen exists as a N_2_ molecule, where two N atoms are bonded through a triple bond. It requires a huge amount of energy to reduce it to NH_3_ (see the equation).

N_2_ + 8H^+^ + 8e^−^ + 16ATP → 2NH_3_ + H_2_ + 16ADP + 16Pi

Chemically, the equation depicts only a direct cost in the form of required ATPs. There is an additional indirect cost to maintain infrastructure of the catalytic process and metabolism of by-product [147]. In a symbiotic nitrogen fixation the required energy is drawn from the host and in a free-living bacterium it is diverted from other cellular processes, which nevertheless, has its consequences. The nitrogenase enzyme is a large complex of MoFe protein (catalytic unit) and Fe protein (reductase unit) components with a size range of 220–250 and 50–60 kD, respectively, depending on the organism [148,149]. The complexity of metal centers for their structure and function makes nitrogenase a unique metalloprotein to understand its mechanism [85,150]. Advances in the mechanism, especially the electron transfer from the Fe protein to the catalytic site of MoFe protein, have been reviewed recently [151]. The nitrogenase complex is encoded by a battery of *nif* genes, which are controlled through an intricate mechanism both at transcriptional and post-transcriptional levels [149,152,153]. Their coordinated expression encoding all the proteins of nitrogenase complex in stoichiometric proportions is essential to assemble a functional holoenzyme, which requires a clear understanding of a regulatory framework of all the genes involved [154]. Nitrogenase is highly sensitive to oxygen, with quick oxidation/inactivation of the catalytic site (MoFe protein) and reductase component (Fe protein) [155]. The reason for the extreme sensitivity of the enzyme to oxygen resides in the evolutionary details of geobiology [156]. The enzyme is insulated from oxygen by structural barriers and through binding of free oxygen by leghemoglobins in legume root nodules [157]. Other free-living bacteria employ different strategies for protection of the nitrogenase from O_2_ and simultaneously meeting a high demand of respiration [158]. Additionally, nitrogen fixation being an energy expensive process is very sensitive to feedback inhibition by an accumulated NH_3_, which requires its efficient removal or assimilation [159]. Together, these factors pose biochemical, genetic engineering and structural challenges to equip the cereals with nitrogen fixing ability.

**Figure 2:**
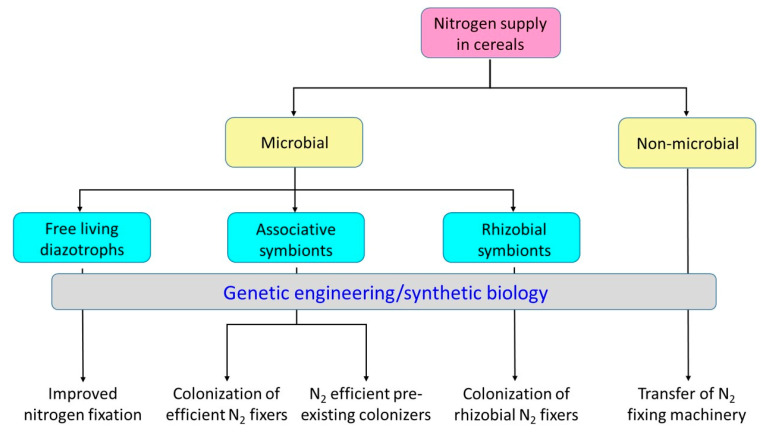
Genetic engineering approaches to improve the availability of biologically fixed nitrogen in cereal crops.

Using molecular biology tools, different approaches have been taken to improve biological nitrogen fixation and supply to cereals (Figure 2). Mostly they were directed at manipulation of microbes harboring nitrogenase activity, but recently the advent of synthetic biology allowed handling of large pieces of DNA or clusters of genes for genetic engineering.

Inoculation of crops with plant growth-promoting bacteria has been studied to enhance yield and production with reduced reliance on chemical N fertilizers [160]. The free-living diazotrophs, e.g., *Azotobacter*, *Beijerinckia*, and *Clostridium*, with the ability to fix nitrogen have been explored in cereals [161,162]. A mutation in the *nifL* gene in *Azotobacter vinelandii* allowed nitrogen fixation at higher ammonium concentration [163]. Manipulations of the glutamine synthetase promoter and the *nifL* gene of this diazotroph not only enabled secretion of high amounts of ammonium but also showed strong proliferation of microalgae and promoted growth in cucumber plants in the absence of added N fertilizer [164]. The deletion in the negative regulatory region of *nifL* genes and inclusion of positive regulatory gene *nifA* in another species of *Azotobacter* enhanced its capability of nitrogen fixation and reduced the reliance on N fertilizer under field conditions of wheat cultivation [162]. The engineered bacterium enhanced the yield by 60% compared to unfertilized controls. These bacteria obtain their energy from decayed organic matter in soil, thus agriculture management practices greatly influence their distribution in soil [165]. Free-living bacteria are an indirect source of nitrogen without host specificity. There are other types of bacteria that closely associate with the host roots and fix nitrogen through a process referred to as associative nitrogen fixation. *Azospirillum*, *Klebsiella*, and *Pseudomonas* are among the bacteria that have an associative relationship with non-legumes including cereal crops [166,167,168]. To enhance nitrogen fixation by the associative symbionts, two biotechnological approaches could be used. Either the pre-existing colonizing bacteria are improved for efficient nitrogen fixation or those with best nitrogen fixing traits are genetically modified to induce colonization in cereals [169]. The authors argued in favor of improving the pre-existing colonizers for nitrogen fixation due to greater complexity involved in the colonization mechanism. The transfer of a gene cluster from *Pseudomonas stutzeri* to the aerobic root-associated beneficial bacterium *Pseudomonas protegens* Pf-5 endowed the strain with a functional nitrogenase, which was able to fix nitrogen and release it as ammonia [170]. Recently, an improved tolerance to ammonia and oxygen by transferring inducible clusters from *P. stutzeri* and *A. vinelandii* has been demonstrated in *P. protegens* Pf-5 [171]. The progress in genetic modifications of associative symbionts has been recently reviewed [144,169].

There is a considerable gap of nitrogen fixation rates from nodules to other types of fixation. The rhizobia fix 50–465 Kg N ha^−^^1^ y^−^^1^, while other associative or free-living nitrogen-fixing species provide 1–170 Kg N ha^−^^1^ y^−^^1^ [144]. Given the large difference in nitrogen derived from different sources, nitrogen fixation similar to legumes, or providing the plant with its own N-fixing machinery are the most desirable outcomes in cereals for reduced or limited dependence on N fertilizers. A symbiotic relationship of rhizobia with legumes evolved over time, where, in nodules, the respiratory requirement of oxygen is sufficiently met with simultaneous protection of nitrogenase from O_2_ [172,173,174]. Non-legumes such as cereals have poor or no symbiotic relationships with rhizobia, although different species from genus *Rhizobium* have been described colonizing cotton, maize, wheat, rapeseed, sugar cane, carrot and rice and a new species *Rhizobium rhizoryzae* sp. nov. from rice has been reported [175]. Some rhizobia were found in cereal plants where expression of the bacterial nitrogenase gene was detected [176]. Apparently, these crops did not develop a shield from high level of oxygen and rhizobia were unable to form specialized structures similar to nodules in legumes [177,178]. The discovery of a nitrogen-fixing cyanobacterium, *Nostoc*, that colonizes intracellular in mucilage-secreting gland cells of *Gunnera* plants points that nodulation is not a pre-requisite for intracellular symbiotic nitrogen fixation [168]. The relationship has been able to satisfy the plant’s nitrogen requirement. Moreover, the rhizobia have been found to fix nitrogen in several non-nodulating legumes [179]. Another example of nitrogen fixation by diazotrophs was found in a unique Sierra Mixe maize landrace that secretes a large amount of mucilage in aerial roots and acquires 29–82% of the required nitrogen through microbiota in mucilage [180]. The further scope of nitrogen fixation through aerial roots in cereals has been discussed in detail [181]. The other novel strains with optimized nitrogenase expression are continuously sought and discovered [182]. Genetic engineering of non-nodulating microbes to expand nitrogen fixation in cereal crops presents another opportunity to meet the nitrogen demand through a symbiotic process. The approach of nodulation in cereal crops by decoding a complete mechanism of a classical rhizobia-legume symbiosis is still being pursued and the work is in progress. The topic highlighting the challenges and building on opportunities has been reviewed [145]. A structural similarity between Nod factors and Myc factors, which activate the signaling pathway during mycorrhizal symbiosis in cereals has been noticed [183]. The strategy of engineering the perception of *nod* factors to activate mycorrhizal symbiosis signaling pathway which in turn can activate the engineered nodulation specific genes has been pursued and the progress made was reviewed [145]. A transgenic rice expressing three legume-specific nodulation (Nod) factor receptor protein genes responded to rhizobial Nod factors and conferred on root hairs the ability to respond to these factors in terms of exhibiting deformations displaying a similarity to initial symbiotic reactions in legumes [184].

The advances in synthetic biology have generated vigorous interest and led to intensified efforts in transferring the nitrogen reduction catalytic machinery to cereal crops. In spite of enormous challenges in this non-microbial or non-symbiotic approach, the developments have been encouraging [144,152]. The selection of a nitrogen assembly site within a plant system is important to address the nitrogen fixation requirements. Chloroplasts and mitochondria were at the forefront when meeting a high energy demand of fixation was taken into consideration. Additionally, these organelles offer a prokaryotic style of gene expression and regulation. The chloroplasts are the active site of O_2_ evolution in the light reaction of photosynthesis. To mitigate nitrogenase sensitivity to O_2_, the expression of *nif* genes during the dark period was contemplated [185]. A window of approximately 4 h of respiratory burst before the onset of dark period can be utilized to express *nif* genes under the rhythmic control of promoters [152]. The expression of active Fe protein by integrating bacterial *nifH* and *nifM* genes into tobacco chloroplasts, but only under a low level of oxygen, has been demonstrated [186]. Further improvements in Fe protein solubility and activity under atmospheric oxygen conditions have been achieved [187]. A significant breakthrough in the stoichiometric challenge of synthetic biology coordinating expression of multiple genes has been reported recently. Yang et al. [188] regrouped 14 essential *nif* genes from *Klebsiella oxytoca* into giant genes either by fusing them together or by expressing polyproteins that are subsequently cleaved with Tobacco Etch Virus protease. The optimal activity of proteins supported *E. coli* growth on dinitrogen. A correct assembly of 15 nif genes (11 genes—*nifB*, *nifH*, *nifD*, *nifK*, *nifE*, *nifN*, *nifX*, *hesA*, *nifV*, *groES*, and *groEL*—from *Paenibacillus polymyxa* WLY78; and 4 genes—*nifS*, *nifU*, *nifF*, and *nifJ*—from *K. oxytoca*) in *Saccharomyces cerevisiae* opened the possibility of a functional nitrogenase complex in eukaryotic hosts [189]. Earlier, 16 *nif* genes were individually targeted to tobacco mitochondria and detectable levels of encoded proteins were observed in the matrix [190].

In the recent past, remarkable progress has been made in various possibilities of biological nitrogen supply or fixation within cereals. The dream conceived in 1970, however, has yet to be realized. Nonetheless, it looks more probable now than in the past that cereal production systems will have less dependence on synthetic nitrogen in future. Assuming successful functioning of nitrogenase in cereals, the task for the scientific community will be far from over. As discussed earlier, conversion of N_2_ into its usable form is an energy or resource-intensive process, and there will likely be negative consequences of N self-sufficiency on plant productivity. Increased photosynthesis and mobilization of photosynthates to root may be a remedial path in that scenario. In conclusion, there will be many unknowns during the improvement of BNF that will require innovative solutions for commercial acceptability of new developments.

## 5. Future Perspectives

Improvement of biological nitrogen fixation for higher nitrogen productivity will increase the sustainability of agriculture production systems. Various strategies have been used to improve the process in an existing symbiotic relationship and widen the scope to non-symbiotic commercial crops. The success of many of such strategies will depend on how well the process of nitrogen fixation is understood. Genomic studies and high-throughput data computing capabilities can play a great role in discerning the underlying mechanism of communication, occupation in the host and nitrogen fixing ability. Equally important is to gain in-depth knowledge of factors responsible for rhizobial persistence in the community of microbiome. The rhizobial genome with some dispensability of genes provides flexibility and opportunity to substitute them with more useful cellular functions. The challenges notwithstanding, many of the genetic traits could be stacked to produce elite strains with overall enhanced performance or for specific environmental conditions. The advent of synthetic biology allows manipulation of large blocks of DNA harboring multiple genes with combined or individual control of gene expression.

Although tremendous efforts over a long time period have resulted in significant progress in different areas of strain improvement (Table 1), the introduction and adoption of such strains in agriculture have been dismally low so far [191,192,193,194]. Many factors that include social, regulatory, environmental and economic considerations are contributing to the lack of application of scientific breakthroughs. One important aspect deserving attention is that most of the studies were confined to lab or controlled environment conditions. The intended effect of such strains under field conditions is unpredictable and their competitiveness with native wild types is unknown [192]. Therefore, rigorous field testing, which requires knowledge of a regulatory framework, appropriate permissions and facility, demonstrating the benefit of elite strains over wild types would greatly help in commercialization of the former. The regulatory constraints of genetically modified microorganisms (GMOs), which are not uniform across different countries, slow down the testing process. To provide a resource for the reader, the US, Canadian and Asian laws regulating GMOs have been reviewed in detail [195,196,197]. In general, there have been concerns on the application of GMOs in food, feed and on native microflora. Irrespective of whether the concerns are unfounded, have scientific merit or simply are a consequence of unknowns, smooth integration of the elite strains in agricultural practices would require parallel studies to allay the fear of GMOs. The available data on the ecological impact of GMOs on native microflora apparently have not raised serious concerns. More studies covering all dimensions of the impact of elite strains will help formulate a strong scientific argument in favor or against. The current advances in high-throughput microbiome and metagenomics analyses are likely to provide a better picture in near future. Molecular biology will continue to play a vital role in the development of elite strains with wide applications that outweigh the risks, if any.

## Figures and Tables

**Table 1:** Modification of rhizobial strains to improve survival and nitrogen fixation characteristics.

Group	Gene	Genotype	Phenotype	Reference
Adhesin biosynthesis	*rapA1*	Overexpression in *Rhizobium leguminosarum*	Increased competitiveness and nodule occupation in red clover.	[20]
Antagonism related	TFX (peptide antibiotic trifolitoxin)	Production in *Rhizobium etli*	Higher rhizosphere competitiveness and nodulation.	[21]
Cellular replication	*parA*	Overexpression in *Azorhizobium caulinodans*	Single swollen bacteroid in one symbiosome, relatively narrow symbiosome space, and polyploid cells were observed when in symbiosis with *Sesbania rostrate.*	[22]
EPS biosynthesis	*pssA* and *rosR*	Overexpression in *R. leguminosarum*	Increased competitiveness and induced more nodules in clover plants.	[23]
	*exoY*	Overexpression in *Sinorhizobium meliloti*	Higher shoot fresh weight and shoot length in *Medicago truncatula*.	[24]
Heat stress	*clpB*	Extra copies in *Mesorhizobium mediterraneum*	Improvement in symbiosis under normal and acidic conditions. Overexpression of *nodA* and *nodC*.	[25]
	*groEL*	Overexpression in *Mesorhizobium*	Improved symbiotic effectiveness in chickpea.	[26]
Hydrogen uptake	*hup*	Gene from *R. leguminosarum* expressed in *Rhizobium tropici* and *Rhizobium freirei*	Increase in nodule efficiency and seed N content in *Phaseolus vulgaris*	[27]
Metal toxicity	*MTL4* and *AtPCS*	Genes from *Arabidopsis thaliana* expressed in *Mesorhizobium hauakuii*	Increased Cd in nodules working on phytoremediation.	[28]
	*MTL4, AtPCS and AtIRT1*		Higher sensitivity and higher accumulation of Cd. Advantage in accumulation of Cu and As.	[29]
	pSinA	Plasmid from *Sinirhizobium* inserted in several *Alphaproteobacteria*	Arsenic resistance and oxidation and heavy metal resistance.	[30]
	*copAB*	Gene from *Pseudomonas fluorescens* expressed in *Sinorhizobium medicae*	Improved root Cu accumulation without altering metal loading to shoots in *M. truncatula.*	[31]
			Improved root Cu tolerance in *M. truncatula*.	[32]
	S-adenosyl-methionine methyltransferase	Gene from *Chlamydomonas reinhardtii* in *R. leguminosarum* bv. *trifolii*	Methylation of arsenite.	[33]
	*ropAe*	Deletion in *R. etli*	Cu tolerance enhanced.	[34]
	*PsMT1* and *PsMT2*	Metallothionein genes from pea expressed in *R. leguminosarum*	Improved tolerance to Cd depicting normal development of nodules.	[35]
Molecular transport	*dctA*	Overexpression *Rhizobium meliloti*	Higher rate of nitrogen fixation in *Medicago sativa.*	[36]
*nif* genes	*nifA*	Gene from *Klebsiella pneumonie* overexpressed in *S. meliloti*	Increased nodulation competitiveness in alfalfa.	[13]
			Did not affect *S. meliloti* competitiveness.	[37]
		Extra copy in *S. meliloti*	Increased alfalfa biomass.	[16]
		Overexpression in *S. meliloti*	Increased nodule formation efficiency and rhizopine synthesis.	[38]
		Overexpression in *Bradyrhizobium japonicum*	Overexpression of *groESL3*.	
		Gene from *K. pneumonie* overexpressed in *Sinorhizobium fredii*	Accelerated nodulation and increased competitiveness in soybean.	[39]
		Overexpression in *S. meliloti*	Improved nitrogen fixing efficiency in *M. sativa*.	[15]
	*nifHDK*	Overexpression in *R. etli*	Increased nitrogenase activity and increased weight and yield in *P. vulgaris*.	[17]
*nod* genes	Random DNA fragment	Random DNA duplication in *R. tropici*	More competitive strains for nodule formation in *Macroptilium atropurprreum.*	[9]
	*nodD1, nodABC and nifN*	Overexpression in *S. meliloti*	Increase in nodulation, nitrogen fixation (acetylene reduction activity) and growth of alfalfa.	[40]
	*nodD*	Overexpression in *R. leguminosarum*	Increased nitrogen fixation in *Vicia sativa* and *Trifolium repens*.	[41]
	*nodD1*		Delayed nodulation and reduced number of nodules on *Vicia* plants.	[42]
	*nodD2*			
	*nolR*	Overexpression in *S. fredii*	Increased EPS production and less number of nodules on *Glycine max*. Increased number of nodules on *Vigna unguiculata.*	[43]
Oxidative stress	*fld*	Gene from *Anaboena variabilis* Overexpressed in *S. meliloti*	Nodule senescence delayed in *M. sativa*.	[44]
			Reduced structural alterations in alfalfa nodules.	[45]
			Less decline in nitrogenase activity under salinity conditions.	[46]
			Improves tolerance to oxidative stress and the survival in the presence of the herbicides paraquat and atrazine.	[47]
	*katB*	Overexpression in *S. meliloti*	Aberration infection thread formation and delayed nodulation on *M. sativa.*	[48]
	*cbb3*	Overexpression in *B. japonicum*	Increase in the symbiotic effectiveness and in O_2_ consumption rate (free-living cultures).	[49]
			Enhancement in symbiotic nitrogen fixation.	[50]
		Overexpression in *R. etli*	Reduced sensitivity of symbiosis with *P. vulgaris* in drought conditions.	[51]
	*vktA* (catalase)	Gene from *Vibrio rumoiensis* expressed in *R. leguminosarum*	Increased N fixation activity into nodules, reduced H_2_O_2_ production.	[52]
	*ahpC*	Overexpression in *Anabaena*	Lowered the peroxide, superoxide and malondialdehyde contents in *Anabaena* strains.	[53]
Phosphate solubilization	*appA*	Gene from *Citrobacter braakii* overexpressed in rhizobia	Increased P content and shoot dry weight of *Vigna radiataradiate.*	[54]
		Gene from *Escherichia coli* overexpressed in *S. meliloti*	Improvement of maize growth in low P soil.	[55]
Phytohormone modulation	*acdS* and *lrpL* (ACC deaminase)	Mutation in *R. leguminosarum*	Decreased nodulation in pea.	[56]
		Genes from *R. leguminosarum* overexpressed in *S. meliloti*	Improved competitiveness, nodulation and shoot dry weight in alfalfa.	[57]
	*iaaM* and *tms2*	Overexpression in *S. meliloti*	Increased number of nodules in *M. truncatula.*	[58]
			Increased tolerance to UV, high salt, low pH and phosphate starvation.	[59]
			Improved nitrogenase activity in nodules and increased stem dry weight.	[60]
			Lower expression of ethylene signaling genes, released larger amounts of P-solubilizing organic acid and lower reduction in shoot dry-weight under P starvation on *M. truncatula.*	[61]
			Induction of many of the transcriptional changes in free-living cells like those occur in nitrogen-fixing root nodule. Increased expression of nitrogen fixation genes and stress response-related genes.	[62]
			Higher tolerance of alfalfa in drought conditions. Higher concentration of Rubisco and lower accumulation of ethylene in drought conditions.	[63]
		Introduction of *iaaM* gene from *Pseudomonas savastanoi* and *tms2* from *Agrobacterium tumefaciens* in *R. leguminosarum*	Fewer number of nodules (but heavier) and increased nitrogenase activity in vetch.	[64]
	*acdS* (ACC deaminase)	Overexpression in *Mesorhizobium loti*	Higher nodulation in *Lotus japonicus* and *Lotus tenuis,* and improved competitiveness of the strain.	[65]
		Gene of *Pseudomonas putida* overexpressed in *Mesorhizobium ciceri*	Stimulated growth and increased nodulation on chickpea under normal and waterlogging stress conditions.	[66]
			Increased nodulation, plant growth and biocontrol potential in chickpeas.	[67]
			Improved growth of chickpea under saline conditions.	[68]
		Gene of *P. putida* overexpressed in *S. meliloti*	Higher biomass of *Medicago lupulina* under copper stress. Enhancement of antioxidant defense system.	[69]
	*ipt* (cytokinin)	*ipt* gene from *Agrobacterium* overexpressed in *S. meliloti*	Increased survival of nodules and increased production of antioxidants under drought conditions in alfalfa.	[70]
	*miaA* (cytokinin)	Mutation in *Bradyrhizobium*	Faster nodule formation and alteration of size and number of nodules in *Aeschynomene.*	[71]
Polysaccharide biosynthesis	*celC*	Overexpression in *R. leguminosarum*	Reduction in biofilm formation, aberrant infection behavior, delay in nodulation and decreased root attachment in *T. repens..*	[72]
		Gene from *R. leguminosarum* overexpressed in *S. meliloti*	Delay in nodulation in *M. truncatula.*	[73]
Salinity and drought stress	*putA*	Overexpression in *S. meliloti*	Increased competitiveness in alfalfa plants under drought stress.	[74]
	*betS*		Rapid acquisition of betaines and better maintenance of nitrogen fixation in salinized alfalfa.	[75]
	*otsA*	Overexpression in *R. etli*	Improved number of nodules, nitrogenase activity and biomass in *P. vulgaris*. Plants recovered from drought stress.	[76]
		Gene from *S. meliloti* overexpressed in *M. ciceri*	Increased growth in saline media. Improved nodules formation and shoot biomass accumulation in chickpea growing in presence of NaCl.	[77]
Siderophore production	*fegA*	Gene from *B. japonicum* expressed in *Mesorhizobium* sp.	Increased growth and nodule occupancy in peanut plants.	[78]
	*fhuA*	Gene from *B. japonicum* expressed in *Rhizobium* sp.	Increased growth and nodule occupancy in pigeon pea.	[79]
		Gene from *E. coli* overexpressed in *Rhizobium* ssp.	Increased nodulation and growth in pigeon pea.	[80]

## Data Availability

Not applicable.
